# Rare *SRRM2* mutation in neurodevelopmental disorders involving hyperphagia triggering severe obesity and other complication

**DOI:** 10.3389/fmed.2025.1492851

**Published:** 2025-02-19

**Authors:** Si-Hua Chang, Xia Wang, Jie-Yuan Jin, Qin Wang, Li-Ping Wu

**Affiliations:** ^1^Department of Hand and Microsurgery, Xiangya Hospital, Central South University, Changsha, China; ^2^School of Life Sciences, Central South University, Changsha, China; ^3^National Clinical Research Center for Geriatric Disorders, Xiangya Hospital, Central South University, Changsha, China; ^4^Department of Pediatrics, Xiangya Hospital, Central South University, Changsha, China; ^5^Department of Nephrology, Xiangya Hospital, Central South University, Changsha, China; ^6^Longgang District Maternity & Child Healthcare Hospital of Shenzhen City (Longgang Maternity and Child Institute of Shantou University Medical College), Shenzhen, China

**Keywords:** *SRRM2*, neurodevelopmental disorders, hyperphagia, obesity, intellectual disability

## Abstract

*SRRM2* encodes a nuclear protein, with functions in pre-mRNA splicing and the formation of nuclear speckles via liquid-liquid phase separation. Despite its critical role in cellular function, the association between *SRRM2* and neurodevelopmental disorders is not well-understood. In this study, we reported a case of a patient exhibiting developmental delay, intellectual disability, delayed language development, facial dysmorphism, macrocephaly, short hands and feet, hyperphagia, and hypotonia, which are similar to the characteristics of previously reported cases of SRRM2-associated neurodevelopmental disorders. Notably, the patient became overweight and subsequently developed several obesity-related complications due to uncontrolled hyperphagia. Employing whole exome sequencing (WES) and Sanger sequencing, we identified a novel missense mutation in *SRRM2* (NM_016333: c.4661A > T, p.Q1554L). This mutation is classified as “Likely Pathogenic” based on the American College of Medical Genetics and Genomics (ACMG) guideline. Overall, this study contributes to the expanding spectrum of known mutations in *SRRM2*, enhances our understanding of its clinical implications, and offers crucial data for the diagnosis and management of affected individuals.

## Introduction

The *SRRM2* gene encodes the nuclear protein Serine/Arginine Repetitive Matrix 2 (SRRM2), which features an conserved N-terminal RNA recognition motif and a region with at least 40% serine and arginine (1, 2). SRRM2 and related proteins form an important complex within the spliceosome, facilitating pre-mRNA splicing and enhancing interactions with splicing factors (3, 4). Meanwhile, this protein acts as a core component in nuclear speckles (2, 5). Its structural domains, rich in arginine and serine, possess the unique physical property of being prone to liquid-liquid phase separation, which drives the assembly of nuclear speckles, a membrane-less organelle (6). Nuclear speckles are currently considered a dynamic nuclear structures involved in complex RNA metabolic steps in the nucleus (7, 8). Proteins crucial for gene transcription and mRNA maturation processes have been localized in nuclear speckles (9). Moreover, SRRM2 also plays an important role in proper cellular and developmental processes during embryogenesis, as highlighted by studies in mouse and C. elegans models (10, 11). These studies emphasize its potential relevance to neurodevelopmental phenotypes.

SRRM2-related neurodevelopmental disorders present with clinical features of mild developmental delay, intellectual disability, speech delay, generalized hypotonia, weight gain, and facial malformations (12). Previous studies have identified an enrichment of *SRRM2* mutations in neurodevelopmental disorders (13). These mutations have been observed with an estimated prevalence of 1 in 11,000 to 1 in 50,000 individuals in the general population (14). However, the accuracy of these figures is subject to verification, given the inherent limitations of cohort studies. Despite these uncertainties, the consistent discovery of pathogenic mutations in SRRM2 highlights its role as a significant genetic factor in neurodevelopmental disorders.

In this study, we report a case of a patient with a neurodevelopmental disorder carrying a novel missense mutation in *SRRM2* (NM_016333: c.4661A > T, p.Q1554L), whose various symptoms are consistent with other case reports of SRRM2-related neurodevelopmental disorders. This report aims to expand the spectrum of *SRRM2* mutations and provide some valuable information for the diagnosis and treatment of patients with SRRM2-related neurodevelopmental disorders.

## Materials and methods

### Subject

The research protocol was approved by the Review Board of the Xiangya Hospital of Central South University in China (2023111889) and informed consent was obtained from all participants. The proband and his family members participated in this study, donated their blood, and consented to the publication of clinical details.

### Whole-exome sequencing

Genomic DNA was extracted from peripheral blood using the DNeasy Blood and Tissue Kit (Qiagen, Valencia, United States), and the quality of the DNA was verified by NanoDrop before being submitted to Berry Genomics (Chengdu, China) for whole-exome sequencing services. The detailed workflow is as follows: Genomic DNA (50 ng) was fragmented to ∼200 bp using enzymatic digestion, followed by end repair and A-tailing at the 3′ ends. Barcoded sequencing adaptors were ligated to the fragments, and ∼320 bp fragments were selected using XP beads. Target enrichment was performed using the NanoWES Human Exome V2.0 kit (Berry Genomics, Beijing, China) according to the manufacturer’s protocol. The enriched libraries were amplified by PCR, purified, quantified by qPCR, and assessed for size distribution with an Agilent Bioanalyzer 2100. Sequencing was conducted on the Illumina NovaSeq 6000 platform (150 bp paired-end mode). Raw data were generated using CASAVA v1.82. The sequencing reads were aligned to the human reference genome (hg38/GRCh38) using Burrows–Wheeler Aligner tool and PCR duplicates were removed by using Picard v1.57^1^. This was followed by bioinformatics studies covering aspects such as mutation detection, the use of standard filtering methods and annotation using tools. Annotation tools mainly included: MutationTaster^2^ for predicting variant pathogenicity, PolyPhen-2^3^ and SIFT^4^ for assessing the potential functional impact of amino acid substitutions, DANN^5^ for scoring deleteriousness, GnomAD^6^ and CMDB^7^ for population frequency data. Further clinical correlation was explored using OMIM^8^. Variants were confirmed for their pathogenicity classification in adherence to the standards and guidelines of the American College of Medical Genetics and Genomics (ACMG).

### Variant validation

Sanger sequencing was used to validate the variants after common filtering. With the help of Integrated DNA Technologies^9^, specific primer pairs (F: CATCTAGAGGGAGAAGCGAATG; R: GGTTCAGGAGAGGAATCAGAAC) were designed for PCR amplification. 2 × Hieff^®^ PCR Master Mix for PAGE (Yeasen, Shanghai, China, 10160ES03) was used to prepare PCR system. The PCR thermocycling conditions were as follows: initial denaturation at 95°C for 3 min, followed by 35 cycles of denaturation at 95°C for 30 s, annealing at 55°C for 30 s, and extension at 72°C for 1 min. A final extension was carried out at 72°C for 5 min. The amplified PCR samples were analyzed using the ABI 3100 Genetic Analyzer (Thermo Fisher Scientific, Waltham, United States).

### Bioinformatics predictions

To assess the functional implications of the identified *SRRM2* mutation, a hydrophobicity prediction was conducted using Protscale^10^. SRRM2 amino acid sequences of various species were downloaded from NCBI^11^. We used MUpro^12^ to predict protein stability and NetPhos - 3.1^13^ to predict phosphorylation modifications.

## Results

### Case description

**FIGURE 1:**
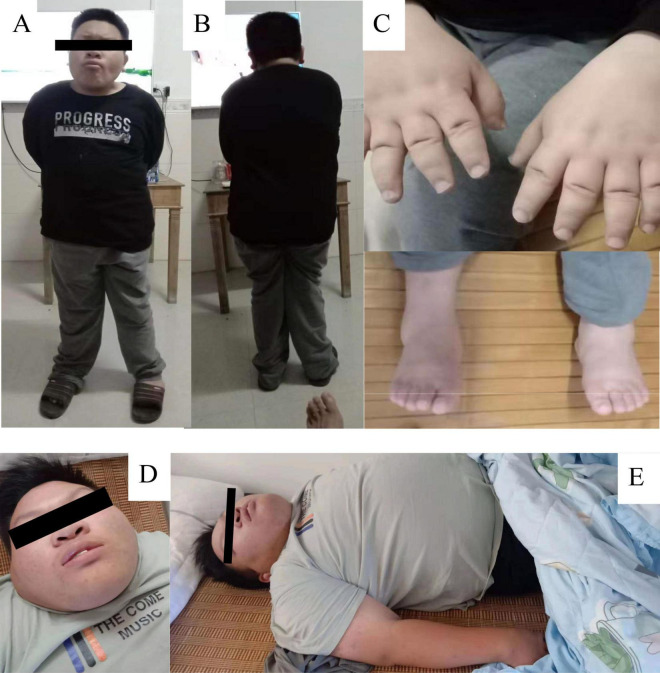
Patient Phenotypic Characteristics. **(A–C)** Display patients at the age of seven, highlighting features of facial dysmorphism, small hands and feet, and hypotonia. **(D,E)** Show the same individuals at age 17, with an emphasis on severe obesity. The images emphasize the clinical manifestations associated with *SRRM2* mutation and neurodevelopmental disorders.

The proband was admitted to our hospital at the age of seven and underwent a comprehensive physical examination. The examination identified facial dysmorphism, short hands and feet, and hypotonia (Figures 1A–C). Further assessments revealed associated neurodevelopmental disorders, including language delay, intellectual disability, and developmental delay. An interview with the parents suggested hyperphagia as an associated phenotype, leading to recommendations for strict dietary management. Despite these recommendations, the primary guardians’ inadequate adherence to the dietary guidelines led to rapid weight gain, resulting in morbid obesity and related health complications over the years.

**FIGURE 2:**
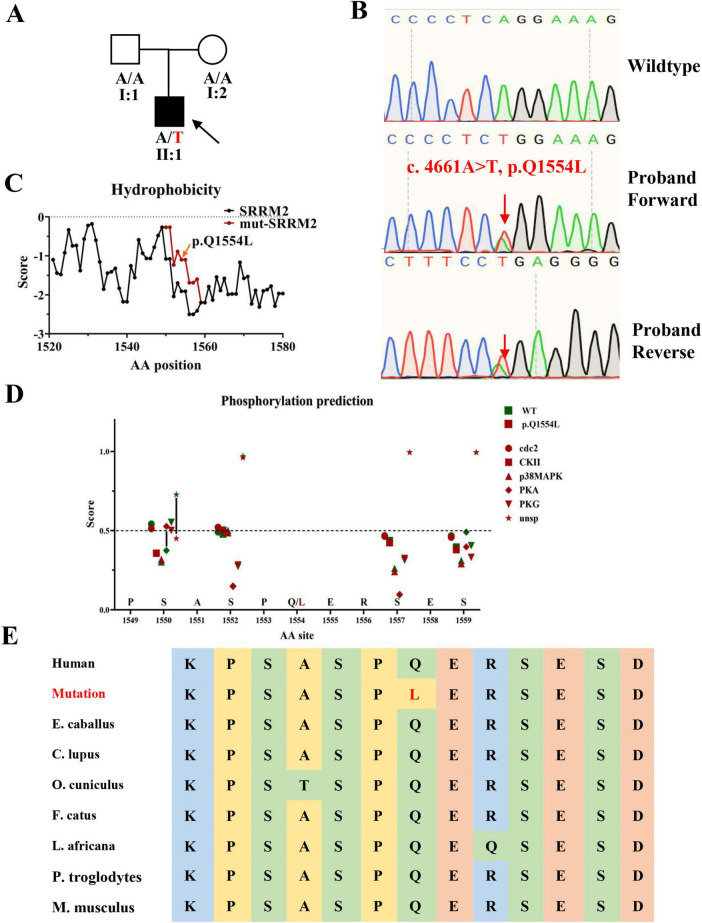
Pedigree, Sanger sequencing, hydrophobicity prediction, post-translational modifications prediction and conservation analysis. **(A)** Family pedigree of *SRRM2* mutation case **(B)** Sanger sequencing results confirming the *SRRM2* mutation in the proband, with the red arrow marking the mutant site. Sequencing was performed using primers designed via Integrated DNA Technologies (IDT) and analyzed using the ABI 3,100 Genetic Analyzer. **(C)** Hydrophobicity prediction of the mutated region, conducted using the ProtScale tool, showing alterations in hydrophobicity that may affect protein function. **(D)** Hydrophobicity prediction of the mutated region, conducted using the NetPhos-3.1 tool. **(E)** Conservative prediction of mutant site among mammal.

At 17 years old, the patient was hospitalized for respiratory distress due to a common cold. At this time, his weight was 103 kg, height 152 cm, and he had a body mass index (BMI) of 44.6, indicative of severe obesity (Figures 1D, E). This hospitalization led to the diagnosis of cardiogenic insufficiency, classified as stage IV cardiac function, along with stage I hypertension and hyperlipidemia. The clinical picture was further complicated by the identification of sleep apnea syndrome. Upon tracing his family history, it was found that neither parent (I:1, I:2) exhibited a similar phenotype (Figure 2A). The timeline of the patient’s case is summarized in Table 1.

**TABLE 1:** Timeline of the case report.

Age	7 years	12 years	17 years
Key events	Initial admission and physical examination	Mucopolysaccharidosis suspected	Hospitalization for respiratory distress
Clinical findings	Facial dysmorphism, short hands and feet, hypotonia, obesity; neurodevelopmental disorders (language delay, intellectual disability, developmental delay)	Phenotypic overlap observed	Severe obesity (weight: 103 kg, height: 152 cm, BMI: 44.6); cardiogenic insufficiency, hypertension, hyperlipidemia, sleep apnea
Genetic analysis	Not yet performed	Genetic testing initiated (WES)	*SRRM2* mutation identified

### Genetic analysis

At 12 years of age in 2019, the diagnosis of mucopolysaccharidosis was suspected for the patient. Whole exome sequencing was performed to investigate relevant genes, including *IDUA*, *SGSH*, *NAGLU*, and *HGSNAT*, which were selected based on previously reported disease-causing genes associated with similar symptoms (11). However, no pathogenic or suspected pathogenic mutations were identified in these genes. Retrospective analysis of our exome data 2 years later revealed a novel missense mutation in *SRRM2*.

To assess the rarity of this mutation, we cross-referenced our findings with the GnomAD and CMBD databases, focusing specifically on mutations with a frequency below 0.001. The pathogenicity of the identified mutations was predicted using bioinformatics tools such as MutationTaster, Polyphen-2, SIFT, and DANN. We focused our analysis on genes containing mutations predicted to be pathogenic or likely pathogenic, especially those associated with neurodevelopmental disorders. In the final stages of our investigation, we concentrated on the mutation in *SRRM2* (NM_016333: c.4661A > T, p.Q1554L), given its phenotypic similarity to other previously documented pathogenic mutations in *SRRM2*. Sanger sequencing validated the presence of this mutation in the patient (Figure 2B). The prediction of changes in hydrophobicity indicated that the mutation leads to significant alterations in the protein’s local hydrophilicity (Figure 2C). The hydrophobicity analysis revealed that the mutation leads to a notable alteration in the hydrophobic profile of the region, which may influence its liquid-liquid phase separation properties. Since phase separation is sensitive to hydrophobic interactions, this change could disrupt the assembly or dynamics of splicing speckles. Furthermore, we compared the mutated region with the predicted post-translational modifications in SRRM2. The results indicated a reduced potential for unspecific phosphorylation of the serine at the 1550^th^ amino acid site (close to the mutation site), while the potential for PKA-mediated phosphorylation was enhanced (Figure 2D). The results of amino acid sequence alignment showed that this was a conserved locus among mammal (Figure 2E). Additionally, through MUpro, protein stability was predicted, yielding a score of 0.31893444. The results indicate an increase in protein stability following the mutation, and the long-term accumulation of misfolded proteins may potentially affect the biological function of the related complexes.

In adherence to the standards and guidelines of ACMG, we classified this *SRRM2* (NM_016333: c.4661A > T, p.Q1554L) as “Likely Pathogenic”: (1) This *SRRM2* mutation is *de novo* (PS2); (2) It is absent from controls in CMDB, and is extremely rare in GnomAD (PM2); (3) Multiple bioinformatics tools predicted this variant to be pathogenic (PP3) (Table 2).

**TABLE 2:** Mutation identified in the proband by whole exome sequencing (WES).

Gene	Variant	Pathogenicity prediction	CMDB	GnomAD	OMIM clinical phenotype	American College of Medical Genetics classification
*SRRM2*	NM_016333: exon11:c.A4661T: p.Q1554L	MutationTaster: D Polyphen-2: PD SIFT: - DANN: 0.99	–	0.00006	AD, intellectual developmental disorder	Likely Pathogenic (PS2, PM2, PP3)

D, disease causing; PD, probably damaging; GnomAD, genome aggregation database; CMDB, chinese millionome database.

## Discussion

We identified a novel pathogenic *SRRM2* mutation in a patient with a neurodevelopmental disorder. The patient presents with intellectual disability, speech delay, short hands and feet, hypotonia, facial dysmorphism, hyperphagia-induced obesity, and subsequent cardiovascular complications potentially related to his obesity. Previous reports have noted hyperphagia, while some patients with *SRRM2* mutations (c.4661A > T, p.Q1554L) exhibit feeding difficulties (12). The association between *SRRM2* missense mutations and obesity phenotypes is further supported by data from the UK Biobank PheWAS^14^. This large-scale population study revealed a strong correlation between SRRM2 missense variants and increased body weight and obesity-related traits. These findings align with the proband’s clinical presentation of severe obesity and hyperphagia, suggesting that *SRRM2* missense mutations may contribute to weight regulation and metabolic phenotypes. The comprehensive clinical report highlights the multifaceted nature of the patient’s condition, including both neurodevelopmental and metabolic challenges. The interplay between these factors highlights the need for clinical management to consider genetic and environmental factors, with appropriate attention to lifestyle factors such as diet and weight control. The proband’s inability to adhere to dietary recommendations, leading to excessive obesity and associated health complications. This case illustrates the complex interplay between genetics, lifestyle, and clinical presentation, underscoring the importance of tailored treatments for complex phenotypes.

**FIGURE 3:**
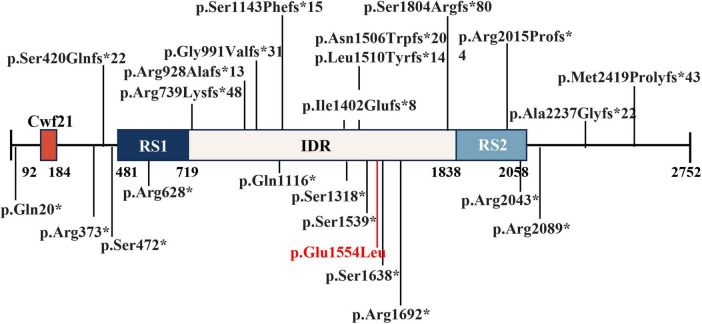
Pathogenic mutations were reported in literature and this report on predicted SRRM2 domains. A total of 36 mutations were reported in literature and report (12, 14, 16). Cwf21: SR-related CTD-associated factor.RS1 and RS2: regions rich in arginine (R) and serine (S) amino acids. IDR: Intrinsically disordered regions.

The assembly of nuclear speckle subregions is driven by SRRM2 phase separation. A recent study focused on the important role played by SRRM2 in mRNA alternative splicing, identifying a dense phase of SRRM2 and SON within nuclear speckles (6). On the other hand, SRRM2 is known for its abundant serine/arginine (SR) repeats, which promote interactions with a variety of splicing factors and RNA-binding proteins. These interactions are crucial for recruiting spliceosomal elements to pre-mRNA sites and assembling the spliceosome, the complex necessary for removing introns from pre-mRNAs. The spliceosome is composed of small nuclear ribonucleic acid (snRNA) and several associated proteins, including small nuclear ribonucleoprotein (snRNP) and non-snRNP factors (15). SRRM2 interacts with these components to facilitate dynamic assembly and disassembly of the spliceosome (4). SRRM2 can influence the inclusion or exclusion of exons in mature mRNAs, which affects gene expression and protein diversity. While SRRM2 is known to play a role in pre-mRNA splicing (6), the specific pathogenic mechanisms underlying the neurodevelopmental disorders observed in our study remain to be fully elucidated. Our mutation (c.4661A > T, p.Q1554L) is located within the intrinsically disordered region (IDR) of SRRM2 (Figure 3), which is known to play a critical role in phase separation and the biogenesis of splicing speckles. This mutation may impact SRRM2’s ability of the phase separation and pre-mRNA splicing, and further studies are needed to verify the function alteration of the mutant SRRM2.

In previous studies, a total of 23 loss-of-function (LOF) mutations in *SRRM2* have been reported as disease-causing mutations, supported by case reports and cohort studies (12, 14, 16). These mutations were associated with neurodevelopmental disorders and other phenotypes, highlighting the role of SRRM2 in neurodevelopmental processes (12, 14, 16) (Figure 3). While the present study focuses on the implications of *SRRM2* mutations in neurodevelopmental disorders, previous research has demonstrated the importance of SRRM2 in embryonic development. For instance, studies in mouse and C. elegans models have shown that SRRM2 is crucial for early developmental processes, including cellular differentiation and organogenesis (10, 11). Given the neurodevelopmental phenotype observed in our patient, it is plausible that the identified mutation disrupts SRRM2 functions that are critical for embryonic development. Although the exact mechanisms remain unclear, this highlights the need for further investigation into the developmental consequences of *SRRM2* mutations. The patient’s phenotype aligns closely with that of other reported patients with SRRM2-related neurodevelopmental disorders. Therefore, we believe that this mutation is the cause of the patient’s disease.

Currently, there are limited studies and reports on the association between SRRM2 and neurodevelopmental disorders. The identification of neurodevelopmental disorders requires precise diagnostic criteria due to the diagnostic difficulties involved. Neurodevelopmental disorders related to *SRRM2* exhibit significant similarities with other conditions, such as mucopolysaccharidosis heightening the risk of misdiagnosis. Considering these factors, there is a strong case for commencing a more extensive cohort study. The study would be to extend phenotype profiles linked to *SRRM2* neurodevelopmental disorders and provide a perspective that we should not ignore missense mutations in *SRRM2*. A key focus should be on improving and standardizing diagnostic criteria to reduce the risk of misdiagnosis. This approach addresses the need for a more accurate understanding of the prevalence and characteristics of these disorders. It also lays the groundwork for developing targeted therapeutic interventions. Advanced genomic sequencing technologies will be crucial in distinguishing SRRM2-related disorders from phenotypically similar conditions, facilitating more accurate diagnoses (17).

## Conclusion

Our study reports a novel *SRRM2* mutation (NM_016333: c.4661A > T, p.Q1554L), which was identified in a boy with neurodevelopmental disorders using WES and Sanger sequencing. The mutation was determined to be a likely pathogenic variant based on ACMG guidance and standards. Our description has enhanced the understanding of the phenotypic characteristics of SRRM2-related neurodevelopmental disorders and broaden the spectrum of pathogenic effects attributed to *SRRM2* mutations. This brings us closer to our goal of providing a reference for genetic consultation, prenatal diagnosis, and treatment options for patients with neurodevelopmental disorders.

## Data Availability

The original contributions presented in this study are included in this article/supplementary material, further inquiries can be directed to the corresponding author.

## Footnotes

1
http://picard.sourceforge.net/

2
http://www.mutationtaster.org

3
http://genetics.bwh.harvard.edu/pph2

4
http://provean.jcvi.org/index.php

5
https://cbcl.ics.uci.edu/public_data/DANN/

6
https://gnomad.broadinstitute.org/

7
https://cmdb.bgi.com

8
https://www.omim.org

9
https://sg.idtdna.com/Primerquest/Home/Index

10
https://web.expasy.org/protscale/

11
https://www.ncbi.nlm.nih.gov/protein/?term=SRRM2

12
https://ics.uci.edu/∼baldig/mutation.html

13
https://services.healthtech.dtu.dk/services/NetPhos-3.1/

14
https://app.genebass.org/
